# Transferring a Community-Based Participatory Research Project to Promote Physical Activity Among Socially Disadvantaged Women—Experiences From 15 Years of BIG

**DOI:** 10.3389/fpubh.2020.571413

**Published:** 2020-09-24

**Authors:** Annika Herbert-Maul, Karim Abu-Omar, Annika Frahsa, Anna Streber, Anne K. Reimers

**Affiliations:** ^1^Department of Sport Science and Sport, Friedrich-Alexander-Universität Erlangen-Nuremberg, Erlangen, Germany; ^2^Institute of Sports Science, Eberhard-Karls-Universität Tübingen, Tübingen, Germany

**Keywords:** physical activity, low socioeconomic status, ethnic minority, transfer, scaling, community-based participatory research, public health impact, sustainability

## Abstract

**Introduction:** Community-based participatory research (CBPR) is an effective approach to health promotion, especially in relation to socially disadvantaged groups. However, the long-term implementation of CPBR-based projects on a broad scale is often challenging, and research regarding the sustainable transfer of participatory research is lacking. This limits the scaling-up and public health impact of CBPR. Therefore, this study examines the mechanisms utilized to transfer and sustain the BIG project, a multifaceted CBPR project aimed at promoting physical activity among women in difficult life situations.

**Materials and Methods:** Borrowing from the RE-AIM framework, we analyzed project documentation and conducted a reflection workshop to investigate methods of transferring BIG to new sites as well as strategies from researchers to support project implementation and the maintenance of program activities at those sites. Moreover, we analyzed the reasons for discontinuing program activities at some former BIG sites and the costs involved in transferring BIG.

**Results:** Since its establishment in 2005, BIG was transferred to and implemented at 17 sites. As of the winter of 2019, the program activities were maintained at eight sites. The average duration of sites that continue to offer program activities was more than 9 years. Discontinued sites maintained project activities for an average of 4 years. According to the study findings, the extent of scientific support, the provision of seed funding, and the local project coordinator, the person managing the project at the site, all have a significant impact on the sustainability of the transfer. A patchwork of funding agencies was needed to finance scientific support and seed funding in BIG. The transfer of BIG projects accrued annual costs of approximately EUR20,000 per site; however, long-term project implementation resulted in a decline in the annual transfer costs of BIG.

**Discussion and Conclusion:** The sustainable transfer of CBPR is challenging but possible, and increased support of research and seed funding can facilitate long-term transfer. Nevertheless, other factors in the implementation setting are beyond scientific control. With scarce financial resources, researchers need to carefully balance the efforts of the sustainability and transfer of CBPR projects. To address this issue, there is a need for further research into the interrelationship of the sustainability and transfer of CBPR projects as well as increased long-term funding.

## Introduction

The participation and empowerment of targeted persons and groups are essential for successful health promotion. According to the Ottawa Charter for Health Promotion, people can only achieve their fullest health potential if they have influence over their own health determinants (1, 2). Two studies, in particular, describe the possible health promotion effects of participatory interventions on (a) the individual level (e.g., strengthening of self-efficacy, health behavior, and perceived social support) and (b) the community level (e.g., strengthening the awareness of vulnerable groups) (3, 4). Furthermore, some studies have pointed out that participatory approaches foster the sustainability of public health interventions due to the integration of stakeholders' resources, the division of responsibilities, the customization of interventions for intended users and the strengthening of abilities and competences (5–8). A special benefit of participatory interventions lies in their potential to reduce health inequality (4). Inequalities exist because many people of low socioeconomic status have a high need for health promotion, as their often precarious life conditions place further strains on their health. However, different barriers limit their chances of accessing health promotion and prevention (9, 10). Participatory interventions have the potential to reach socially disadvantaged people because they incorporate low-threshold interventions that fit the needs of vulnerable groups (5, 11).

To assess the public health impact of such interventions, RE-AIM, which was conceived by Glasgow et al. (12), was established as an important framework. According to RE-AIM, public health impact is achieved by reaching many people (those who are targeted), through an effective intervention at different sites over time. The public health impact of community-based participatory research (CBPR) projects might be low in many cases, simply because sustaining such projects and transferring them to new sites have been challenging (13–15). Key to achieving the public health impact of CBPR projects is sustainability, since the effects of interventions often only occur years later (16). As such, long-term CBPR interventions are needed to achieve structural changes so as to prevent the inefficient and pointless input of resources and the disillusionment of participants (16). Further, CBPR health interventions need to be transferrable to multiple sites, since it is only in the act of transferring interventions that the potential of reaching more people and settings increases and limited resources are utilized more efficiently (e.g., investments for program development) (17, 18). The transfer and sustainability of CBPR interventions have hitherto proven to be challenging and have not been well studied (8, 16, 18). Ultimately, this has led to a lack of evidence regarding the transfer, sustainability, and public health impact of these interventions (3, 6, 18, 19).

On top of the challenges involved in transferring and sustaining CBPR projects, unfavorable public health structures for funding the transfer of these projects exist in many parts of the world (20–22). For instance, the German healthcare system has been described as having a strong curative, rather than preventive, orientation (23). Recently, a law strengthening prevention and prevention research was passed and implemented (24). The so-called prevention act enhances collaboration between different stakeholders as well as the role of the Federal Center for Health Education (BzgA). However, CBPR health promotion projects had been taking place in Germany before the prevention act was passed. The corresponding projects were funded for limited time periods on a small scale by various actors in the field of health promotion, such as national, federal, and municipal agencies or sickness funds (25–27). In the absence of a national funding agency with the capacity to fund initiatives to vertically “scale-up” (growth of the project's reach) or horizontally “scale-up” (transfer to more sites) such CBPR projects, sustaining and transferring a successful project required a patchwork of funding agencies (22, 26–28). This might have limited the successful transfer and, hence, the public health impact of interventions.

This case study investigates how the BIG project “*Bewegung als Investition in Gesundheit*” (movement as an investment in health), a CBPR project, was transferred and sustained across 17 sites in Germany. BIG is a participatory project aimed at promoting physical activity (PA) among socially disadvantaged women (e.g., of a low socioeconomic status or ethnic minority background). Based on the above-described challenges involved in achieving public health impacts of CBPR projects, the aim of this case study was to examine the mechanisms of successful transfer and sustainability strategies of this CBPR project.

## Methods

### BIG—A CBPR Project for Physical Activity Promotion

The BIG project was initiated in 2005 at the Friedrich-Alexander-University Erlangen-Nuremberg (FAU) and has now been transferred from the first community to 16 other sites. The aim of BIG is to promote PA among socially disadvantaged women. This includes women in difficult life situations, who face challenges due to their migration background, low education, unemployment, welfare dependency, low income, and/or their status as single mothers. For these women, access to PA and sport is often impeded because of barriers such as the lack of culturally sensitive exercise offers, language difficulties, high membership fees, or the lack of childcare services. Their chance of benefitting from the positive outcomes of PA are reduced (29), and evidence suggests that the rate of physically inactive individuals is above average among socially disadvantaged women (30).

To counteract this inequality, the BIG project empowers women to improve their access to PA through a participatory approach. As part of this approach, socially disadvantaged women, researchers, local project coordinators, policymakers, and other local experts (e.g., sport club representatives) jointly plan and implement activities in a number of cooperative planning sessions (31). The diverse composition of the cooperative planning group is essential for the implementation of BIG, as all stakeholders provide different resources (e.g., policymakers can provide funds, and sport associations can provide access to sports facilities). Women articulate their needs in terms of exercise offers and support the implementation of these offers.

### BIG—Reach of Exercise Programs, Attendance, and (Health) Effects

Exercise programs implemented through BIG address existing economic, social, cultural, environmental, and behavioral barriers to participation. This is achieved by lowering attendance fees, offering free child care, having female instructors, choosing facilities close to where women live, and the non-requirement of membership to attend classes. Women advertise the exercise programs by word of mouth. Exercise classes offer a range of activities, from aerobic fitness, Zumba, Nordic walking, and martial arts to women-only indoor-pool hours (29). The number of attendees per class ranges from 5 to 20, depending on the offer. Classes commonly take place once or twice per week. In 2019, more than 800 women regularly participated in about 60 different exercise classes at BIG sites.

Previous analyses of BIG have demonstrated that its exercise classes do indeed reach women in difficult life situations (32). There is also evidence of the health-promoting effects of BIG on women. Women attending BIG exercise classes reported increased social networks and beneficial effects on their physical and mental well-being (29, 32). By taking part in the cooperative planning process, the women reported increased self-efficacy, as they were empowered to voice their interests and increase their knowledge of political and administrative processes (29). On a structural level, BIG improves municipal opportunities for exercise by strengthening local networks for health promotion, establishing routines for citizens to participate in municipal decision-making processes, and removing barriers to participation in exercise for women in general (33).

### Data Collection and Analysis

This case study reports on the efforts to transfer (adopt) and sustain BIG. Regarding the transfer of BIG, we investigated (1) different methods of recruiting new BIG sites and (2) different support strategies for implementation at these sites. Pertaining to the sustainability of BIG, we examined (3) the years, up to the winter of 2019, that BIG was maintained at the sites and (4) the reasons given by sites for discontinuing BIG. Further, we investigated (5) each site's costs regarding the transfer of BIG.

Information on recruitment methods (1) and support strategies (2) was gathered through a data analysis of unpublished project reports and meeting protocols from the last 15 years. This included field notes from informal consultations with current and former local project coordinators and staff at the various municipalities. Over the years, different recruitment methods and support strategies were carried out. Recruitment methods varied from conference presentations and open calls to networking. Support strategies provided by FAU researchers to communities included the drafting of an implementation manual, setting up a system for the phone-based and face-to-face counseling of communities and providing extensive on-site support to municipal staff in charge of the project. In order to inquire as to whether the BIG project was (3) implemented/maintained or (4) discontinued, the municipalities were contacted by phone. To shed light on the reasons why project activities were discontinued at different sites and to validate this information, a reflection workshop was conducted with three researchers who had supervised the BIG project.

Regarding the costs of transfer (5), grant applications were screened to extract information on the amount of funding the FAU received for carrying out and transferring BIG. From these data, the annual funding amount per site was calculated. This was achieved by dividing the funding amount by the duration of the funding phase and the number of new sites. The annual long-term costs of the transfer were also calculated. This was done by dividing the funding amount of each funding phase by the average years of the project's lifespan at the sites on which BIG was implemented during this funding phase.

Costs covered the initial project development and its transfer to other sites. This included staff and travel costs at the FAU to initially develop the project, recruit, and support municipalities willing to implement BIG and evaluate the effects of BIG at the individual and structural levels. For three subprojects (BIG, BIGff, and BIG.kompetenz), funding also included seed money for the communities to set up the project and hire a local project coordinator.

## Results

Since 2005, six subprojects [BIG, BIGff, BIGGER(t), BIG.Manual, BIG.kompetenz, and BIG.Disseminierung] implemented and transferred the BIG approach to 17 sites. As BIGff and BIGGER(t) utilized the same approach, they are reported on jointly. The aim of each subproject was to develop more effective ways of transferring BIG in order to increase its public health impact.

### Recruitment of New Sites for Project Transfer

Table 1 reports on the different recruitment strategies for all the subprojects. Across all the subprojects, most of the sites (*N* = 13) were recruited through open calls for applications. This recruitment strategy was used in four subprojects (BIGff, BIG.Manual, BIG.kompetenz, and BIG.Disseminierung). The researchers directed a standardized letter to all Bavarian municipalities (mayors, offices, and coordinators of sports or integration) with more than 20,000 inhabitants. Three sites were obtained, as the researchers addressed local stakeholders directly. One site (Regensburg) was recruited after presenting the project at a conference.

**Table 1:** Description of the BIG subprojects, funding amounts, and transfer costs.

**Subproject, funding period, and funding agency**		**Support provided to communities**	**Financial support for communities**	**Recruitment method**	**Number of sites recruited**	**Funding amount**	**Annual costs of scientific support per site**	**Annual long-term transfer costs**
1	**BIG** 2005–2008 Research sector at national level; health sector at states level	Researchers coordinated and implemented the project	Project implementation funding	Direct address of sites	1	€561,125.00	€160,321.43	€40,080.36
2	**BIGff** 2008–2009 Health sector at state level	Extensive assistance of local project coordinators	Seed funding for 4 sites	- Project presentation at a conference - Open calls for applications - Direct address of sites	7	€305,410.00	€16,361.25	€37,180.35
	**BIGGER(t)** 2008–2010 Health sector at national level		Not provided					
3	**BIG.Manual** 2009–2011 Health insurance company	- BIG manual, - Phone and face-to-face counseling	Not provided	Open calls for applications	2	€127,200.00	€29,353.85	€28,266.67
4	**BIG.kompetenz** 2012–2015 Health sector at state level; health insurance company	- BIG competence center (offering counseling and network-meetings for all local BIG coordinators) - BIG manual	Seed funding (10 h/week local project coordinator)	Open calls for applications	5	€361,023.00	€18,051.15	€124,490.69
5	**BIG.Disseminierung** 2015–2019 Health insurance company	- Coordinator at state level in Berlin - BIG competence center - BIG manual	Not provided	Open calls for applications in all districts of Berlin	2	€183,203.20	€20,739.98	€122,135.47

Besides the recruitment method, the number of new sites depended on whether the funders, who supported the researchers at the FAU, also provided seed funding for the project implementation at the sites. This seed funding facilitated the decision-making of the local administrators to implement the project as well as the recruiting process of new sites. Seed funding was provided for three subprojects (BIG, BIGff, and BIG.kompetenz). Ten of the 17 BIG sites were recruited when seed funding was provided, which was used to support the setup of the project and the financing of a local project coordinator at the site.

### Support Strategies for Project Implementation

In each subproject, the FAU tested new strategies to support the sites (Table 1). Within the initial subproject (BIG, 2005–2008), the researchers, targeted women, and persons in charge of project implementation collaborated to develop the BIG approach and went on to probe and readjust it in practice. They coordinated the project onsite (e.g., by conducting the cooperative planning process and building a BIG network). After 3 years, the municipality took over the coordination of the project.

From the beginning of the subprojects BIGff and BIGGER(t), each site had its own local BIG coordinator. The objective was to relocate the coordination and strategic planning of the project from the researchers to a responsible person at the site. The researchers provided extensive support to the local project coordinators by holding weekly exchanges, assisting with network-building and moderating the cooperative planning process. In order to reduce the level of assistance and enable local coordinators to conduct the project more independently, the researchers developed a manual (34) in collaboration with the practice partners. This manual—structured in six sections: (1) about BIG, (2) discovery phase, (3) preparation, (4) cooperative planning process, (5) implementation management, and (6) sustainability assurance/networking—was intended to describe every phase of the project implementation in easy-to-understand language and with the use of many examples, practical advice, and working aids (e.g., questionnaires and quality assessment forms). Many practice partners and other researchers reported that the BIG manual was helpful, not only for the implementation of BIG but also for other participatory projects. Nevertheless, in addition to the BIG manual, the local project coordinators needed consultation. In order to provide implementation support and facilitate the transfer of BIG, the so-called BIG competence center was established at the FAU in 2012. Funded by different sources, and staffed with a 0.5 researcher position, the BIG competence center consulted sites in implementing and planning, evaluated BIG at the sites, and initiated further transfer of the project.

### Maintenance of Program Activity at the Sites

**Figure 1:**
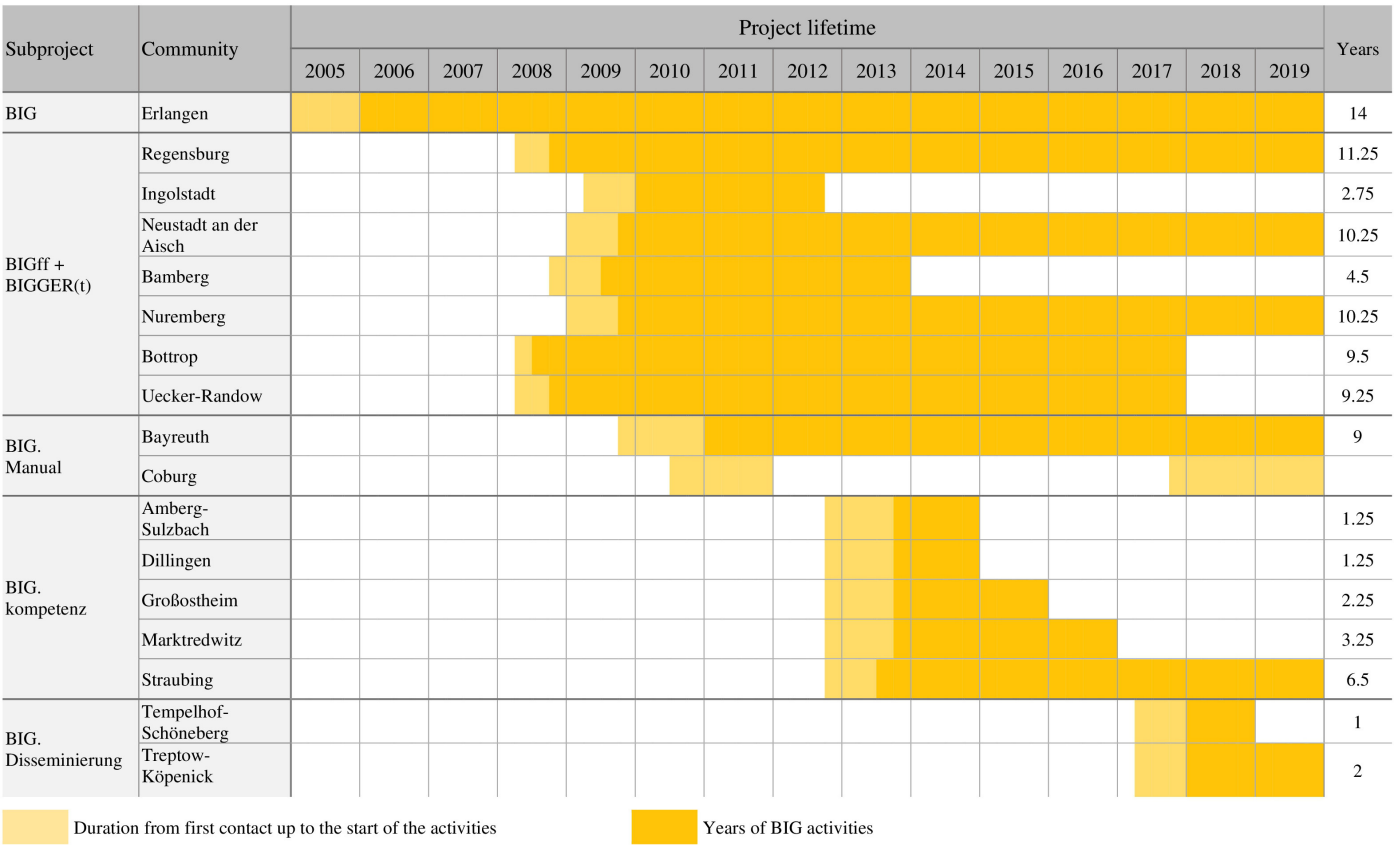
Duration of project activity per site.

At seven of the sites, BIG courses were being offered up to the winter of 2019 (Figure 1). The projects' lifespan at these seven sites was, on average, 9.8 years. Another site restarted the implementation process of BIG following an initial unsuccessful attempt (Coburg). The duration of the project activities at each site is shown in Figure 1. The first BIG site (Erlangen) recorded the longest lifespan (14 years). There, about 30 PA courses were offered per semester. Four sites offered courses for at least 9 years (the number of PA courses is between 2 and 11).

At the nine sites that had not maintained program activities, the projects' lifespan averaged 4.2 years. Two of these sites kept the project running for over 9 years before it came to an end. Four sites offered PA courses for 1–2 years.

### Reasons for Discontinuing BIG at the Sites

The most common underlying reason for the termination of the BIG project was, in one way or another, connected to the local project coordinator. This was the case at six of the 10 sites where project activity was discontinued. Two of these sites had to terminate the BIG program because the local coordinator retired, and no successor could be found. At four sites, the local project coordinators could not continue to manage the project due to other work tasks, maternity leave, or extended sick leave. However, at two sites where local project coordination changed, the handover of responsibilities was successful. Furthermore, five local coordinators were incapable of building a strong network due to a lack of qualified and relevant contacts within their reach. Three sites stated additional reasons for the termination of the project, including insufficient financial resources and funding. This could be linked to the low-fee courses being offered, for which external funding support through the municipality or other entities would have been necessary. Two sites further mentioned that they did not have sufficient participants or trainers for the courses. At some sites, a combination of the aforementioned issues presaged the abandonment of the project.

### Project Transfer Costs

The amount of funding depended on the timespan of the funding arrangement, the extent of scientific support, and the provision of seed funding. Table 1 presents an overview of the funding amounts and transfer costs of each subproject.

Funding for the first subproject was, by far, the highest, and thus the scientific support provided to the first site resulted in high annual costs. The largest expenditure was the cost of personnel relating to the development, evaluation, and implementation of the project. Given this site's long lifespan, the annual long-term transfer costs were not exceptionally high. The lowest annual costs per site were recorded for the subprojects BIGff + BIGGER(t). Even though the total funding amount for these subprojects was rather high, it was split between many new sites (*N* = 7), thus reducing the per-site costs. The funding amount and long-term transfer costs of the BIG manual were the lowest. Nevertheless, the annual costs covering support per site were the second highest, as only two sites could be recruited for this subproject. For BIG.kompetenz and BIG.Disseminierung, the long-term transfer costs were striking. These high costs did not result from actual higher transfer-related expenditures. Instead, they resulted from the more recent start of the subprojects at these sites, which meant that the transfer costs could not yet be amortized.

It is apparent that more support [for BIG, BIGff + BIGGER(t)] did not automatically result in higher annual costs per site. The costs depended on the extent of support, the timespan of funding, and the number of new sites during the funding phase. Although these three factors varied in each subproject, the annual cost of support from the researchers was approximately EUR20,000 per site. The annual long-term transfer costs of BIG and BIGff + BIGGER(t) show how a rather high amount of funding can amortize through sustainability of program implementation.

## Discussion

Over the years, the transfer of BIG to 16 other communities yielded several valuable experiences: (1) in order to recruit new communities, BIG mainly utilized open calls for applications and contacting municipal administrations. This likely resulted in the participation of communities with a genuine and intrinsic interest in the BIG project. Of those, about 35% were able to sustain BIG over time, with an average lifespan of 9.8 years. In other communities, the BIG project ceased, on average, after 4.2 years. In one municipality, the transfer did not result in the promotion of PA activities, and thus, the project failed to implement. (2) Due to the German healthcare system's (scarce) resources for conducting CBPR (28), BIG was forced to rely on a patchwork of funding agencies and funding schemes to transfer the project. In some subprojects, external seed funding for communities was budgeted to stimulate the uptake of BIG. This scarcity of resources made the researchers focus on effective ways to transfer BIG—a quest that is still ongoing. (3) These efforts resulted in different approaches in terms of how to support communities in implementing a CBPR project. The experiences portrayed in this study exemplify that, in addition to seed money, hands-on support in the local implementation of BIG was essential. The implementation of BIG using the manual-only approach, while highly cost-effective (lowest funding amount and lowest long-term transfer costs), did present some limitations. However, the manual proved to be useful when the communities also received additional support from researchers. This study's tentative results suggest that more support from researchers (e.g., by counseling local project coordinators on whom to invite to the planning process as well as attending the planning process) had a positive influence on the sustainable implementation of the projects and did not necessarily incur higher costs in the long run. (4) Aside from the methodology employed in this study, vital factors beyond the researchers' control also played a determining role in the success of project transfers. The two main reasons for the termination of BIG in some communities was the resignation or retirement of the local coordinator and the impossibility of finding a sufficient number of supportive stakeholders or key community members to advertise the project to targeted persons. According to these results, a local project coordinator and his or her competences and capacities are of fundamental importance for the project's success. To facilitate successful handovers of responsibility, a strong network supporting the implementation of BIG appears favorable. (5) Consequently, this study identified the following necessary elements within local coordinators' set of competences and capabilities to facilitate a successful transfer: (a) a local BIG coordinator who already has relevant stakeholder contacts; (b) knowing how to network with the target group, relevant stakeholders, and policymakers; (c) having the scope and opportunities to act (i.e., time, funds, etc.) and the competences to network and implement offers; and (d) being able to draw on support from experienced CBPR researchers. These elements correspond to the results of other studies on the role of program champions (those advocating for the program and its continuation) and their capacity to act (e.g., funding and community/stakeholder support/involvement) (8, 19, 35).

### Transferring and Sustaining CBPR Interventions

A major challenge in achieving public health impacts through CBPR is the sustainability of a local CBPR project and, often simultaneously, transferring it to other communities. As such, researchers face two conundrums: How do we transfer a CBPR intervention that is, by nature, highly context-bound? How can we balance efforts to sustain a project at one site with efforts to transfer it to other sites? Based on the experience in BIG, we suggest that a sustainable transfer requires strong and individual support from municipal administration staff in order to adapt the intervention to the implementation setting and, thereby, increase the likelihood of a sustained implementation. Achieving a public health impact requires researchers to work with communities to “normalize” CBPR projects, i.e., incorporating it in routine practice (36). Under scarce financial resources, researchers need to balance the efforts needed to sustain CBPR interventions. This means that the scientific support is limited in time, and the number of new sites that can receive this support is restricted. This, in turn, limits the transfer of the intervention on a broader scale. Therefore, long-term funding mechanisms are needed (21) for the sustainable transfer of evidence-based and successful health promotion interventions. In BIG, this issue was overcome by utilizing a patchwork of funding agencies to transfer the project to other sites. The current development of the German public health funding system provides an opportunity to establish structures that strengthen the long-term funding of research transfer and, thus, foster the public health impact of research. In addition to the scientific influence, there are a number of factors in the implementation setting that are beyond the control of researchers, which appear to partially determine the success of transferring CBPR, as other models on scalability have demonstrated (14, 37).

To cope with the challenging process of transferring and sustaining CBPR, there is a need for more research into the interaction and interdependence between sustainability and transfer. There are studies, frameworks, and models that depict sustainability as an indicator of a successful scale-up (14). However, this disregards the influence of sustainability on subsequent horizontal and vertical scale-up. For instance, it is only through long-term implementation that interventions can enfold their potential reach (vertical scale-up) and demonstrate effectiveness in increasing the prospect of their transfer to additional sites (horizontal scale-up) (38). Moreover, such research could help identify factors that similarly foster transfer and sustainability (e.g., a competent local coordinator).

### Limitation and Strengths

Some study limitations should be considered. This case study focused on the recruitment of new sites, support strategies, the maintenance of program activity, reasons for discontinuing program activity, as well as transfer costs as the sole relevant factors in the sustainable transfer of the BIG project. As they can have a huge impact on sustainability (8, 14), it would be interesting and meaningful if future research could investigate contextual factors relating to the implementation setting, such as political support or external funding for a CBPR project.

Furthermore, this case study focused on the cost of transfer and included expenses relating to research and seed funding. To determine the cost of the project's implementation, further research should consider funding from municipalities and other financial contributions from external parties. Moreover, we examined the BIG project as an example of sustainably transferred CBPR. Hence, only data from one project were included. Therefore, it cannot be said with certainty that the results are representative of other CBPR projects. Future research on the sustainability and transfer of CBPR projects is required in order to identify strategies that support the sustainability and transfer of evidence-based interventions and maximize their public health impact.

## Conclusion

Although the sustainable transfer of CBPR is challenging, the BIG project was successfully transferred to 16 sites over the last 15 years. This study identified factors in the implementation setting that are of central significance for sustainable transfers and, thus, for the public health impact of CBPR, which are beyond the control of research (capacities of local coordinators, funding stability, and the support of local networks). Therefore, it might be necessary to examine contextual factors and, where needed, to appropriately focus on further developing the requirements in the setting. However, other factors within the control of researchers do facilitate sustainable transfers. In BIG, the high extent of scientific support for the sites, seed funding, and open calls for applications positively influence the long-term transfer of the project. To foster the public health impact of interventions, we recommend more research on the interrelationship of sustainability and transfer as well as increased long-term funding for research on sustainably transferring effect-proven interventions on a broad scale.

## Data Availability Statement

The data generated and analyzed in this study is available upon reasonable request from the corresponding author annika.herbert-maul@fau.de.

## Ethics Statement

Ethical review and approval was not required for the study on human participants in accordance with the local legislation and institutional requirements. The patients/participants provided their written informed consent to participate in this study.

## Author Contributions

AH-M collected, analyzed, and interpreted the data and drafted the manuscript. KA-O participated in the interpretation of the data and helped in the drafting and revision of the manuscript. AF and AS participated in the data collection. AF, AS, and AR critically reviewed and revised the manuscript. All authors read and approved the final manuscript.

## Conflict of Interest

The authors declare that the research was conducted in the absence of any commercial or financial relationships that could be construed as a potential conflict of interest.

## Abbreviations

BIG: *Bewegung als Investition in Gesundheit* (movement as an investment in health)
CBPR: community-based participatory research
PA: physical activity
RE-AIM: reach, efficacy, adoption, implementation, maintenance.
